# Identification of Bacterial Blight Resistant Rice Seeds Using Terahertz Imaging and Hyperspectral Imaging Combined With Convolutional Neural Network

**DOI:** 10.3389/fpls.2020.00821

**Published:** 2020-06-24

**Authors:** Jinnuo Zhang, Yong Yang, Xuping Feng, Hongxia Xu, Jianping Chen, Yong He

**Affiliations:** ^1^College of Biosystems Engineering and Food Science, Key Laboratory of Spectroscopy, Ministry of Agriculture and Rural Affairs, Zhejiang University, Hangzhou, China; ^2^State Key Laboratory for Managing Biotic and Chemical Threats to the Quality and Safety of Agro-products, Key Laboratory of Biotechnology for Plant Protection, Ministry of Agriculture and Rural Affairs, Zhejiang Provincial Key Laboratory of Biotechnology for Plant Protection, Institute of Virology and Biotechnology, Zhejiang Academy of Agricultural Sciences, Hangzhou, China; ^3^State Key Laboratory for Managing Biotic and Chemical Threats to the Quality and Safety of Agro-products, Key Laboratory of Biotechnology for Plant Protection, Ministry of Agriculture and Rural Affairs, Zhejiang Provincial Key Laboratory of Biotechnology for Plant Protection, Institute of Plant Virology, Ningbo University, Ningbo, China

**Keywords:** terahertz imaging technology, near-infrared hyperspectral imaging technology, rice bacterial blight, convolutional neural network, seed identification

## Abstract

Because bacterial blight (BB) disease seriously affects the yield and quality of rice, breeding BB resistant rice is an important priority for plant breeders but the process is time-consuming. The feasibility of using terahertz imaging technology and near-infrared hyperspectral imaging technology to identify BB resistant seeds has therefore been studied. The two-dimensional (2D) spectral images and one-dimensional (1D) spectra provided by both imaging methods were used to build discriminant models based on a deep learning method, the convolutional neural network (CNN), and traditional machine learning methods, support vector machine (SVM), random forest (RF), and partial least squares discriminant analysis (PLS-DA). The highest classification accuracy was achieved by the discriminate model based on CNN using the terahertz absorption spectra. Confusion matrixes were pictured to show the identification details. The t-distributed stochastic neighbor embedding (t-SNE) method was used to visualize the process of CNN data processing. Terahertz imaging technology combined with CNN has great potential to quickly identify BB resistant rice seeds and is more accurate than using near-infrared hyperspectral imaging.

## Introduction

Rice is one of the most important food crops: more than half the world’s population and 60% of the Chinese population relies on it for food (Yuan, 2014). Good and stable yields of rice are therefore needed for survival and to avoid the problems of food shortage. According to some research, the world population will reach 10 billion by 2050 requiring food production to increase by 60–100% (Jaganathan et al., 2018). However, a number of rice diseases seriously affect rice yield and quality over large areas (Shasmita et al., 2019). Rice BB, caused by *Xanthomonas oryzae pv. oryzae* (Xoo), is one of the most destructive diseases in all the major rice growing countries, typically reducing yields by 20–30%, but by as much as 50% in years when the disease is prevalent (Han et al., 2014). Chemical control of the disease is temporarily effective but often leads to long-term environmental pollution. It is generally recognized that the most environmental-friendly, safest and effective strategy for disease control is to breed and deploy resistant cultivars, and this depends on the identification and functional study of resistance genes. Chromosome single Segment Substitution Lines (CSSLs) can be constructed by introducing the quantitative trait loci (QTLs) for disease resistance into rice under the assistance of molecular makers. Nevertheless, even when QTLs for rice disease resistance have been identified, it still takes a lot of time and labor to select suitable rice seeds for further breeding. It would therefore be a great help if plant breeders had an easy and reliable method to quickly identify those rice seeds that contain the desired QTLs for disease resistance.

Terahertz spectroscopy uses terahertz radiation, electromagnetic waves with a frequency between 0.1 and 10 Thz (30 μm–3 mm), to do qualitative and quantitative analysis (Liu, 2017). Due to the special position of the terahertz band in the electromagnetic spectrum, the terahertz waves are transient and have high penetration, broadband, coherence, and low energy (Demidova et al., 2016). The terahertz band with a radiant energy level of approximately 1–10 meV corresponds to the low-energy rotational modes or vibration modes of molecules and so provides a means for detecting most biomolecules such as DNA, protein etc (Hu et al., 2017). Terahertz spectroscopy is now widely used in biology (Mueller-Holtz et al., 2014), security (Shen et al., 2005), communication technology (Nagatsuma et al., 2016), and food safety (Qin et al., 2013). Nakajima et al. (2019) investigated the potential of terahertz spectroscopy to monitor and quantify starch in plants and identified a peak in the terahertz spectrum that was attributed to starch. Transgenic rice seeds could be identified with an excellent rate of 96.67% reliability using terahertz spectroscopy imaging technology and without taking much time or using any complicated preprocessing steps (Liu W. et al., 2016). It has also been shown that changes to the concentration of individual rice proteins can be accurately identified (Xu et al., 2015). Near infrared (NIR; 750–2500 nm) spectroscopy has also been used to obtain unique spectral signatures from samples (Feng et al., 2017). The absorption region of relative overtones and combinations of hydrogen-containing functional groups such as C-H, O-H, and N-H is consistent with the absorption region of the NIR spectra (Feng et al., 2017). This makes NIR hyperspectral imaging a powerful tool which is able to simultaneously obtain spatial and spectral information to do qualitative or quantitative analysis. Especially, NIR hyperspectral imaging technology has been used for fast non-destructive testing in various applications, including seed identification, plant and food quality detection (Gutierrez et al., 2018; Femenias et al., 2020; Qin et al., 2020). NIR spectroscopy combined with chemometric analysis to separate CRISPR/Cas9-induced rice mutants from normal rice and found that SVM with a successive projections algorithm (SPA) achieved the best classification performance (Feng et al., 2017). Orrillo et al. (2019) used NIR spectroscopy to identify authentic black pepper in samples adulterated with papaya seeds with the help of partial least squares regression (PLSR). Cui et al. (2019) showed that a method using NIR spectroscopy and PLSR could be used reliably for maize haploid seed screening. And also deoxynivalenol (DON) contaminated wheat kernels were successfully non-destructively classified and quantified with the help of NIR imaging technology using PLSR and LDA (Femenias et al., 2020). Apart from that, fish filet substitution and mislabeling were detected by multiple hyperspectral technologies and the combination of classifiers and spectral dataset had made a great help to choose the best model (Qin et al., 2020).

Different spectroscopy techniques acquire sample information from different levels, leading to complex spectral data. The hidden information in these data can be fully utilized in combination with efficient modeling algorithms. The deep learning algorithm is a rapidly developing method that can extract the characteristics of information autonomously and effectively classify and characterize it. With the rapid development of deep learning algorithm, more and more studies turn the focus on its implement in object detection, image classification and other computer vision field (Guo et al., 2016). Nie et al. (2019) classified hybrid okra and loofah seeds using NIR spectroscopy combined with the deep convolutional neural network (DCNN) and the classification accuracy of different varieties was all above 95%. Liu P. et al. (2016) proposed a classification algorithm based on active learning of deep networks for hyperspectral images. Mou et al. (2017) also proposed a novel recurrent neural network (RNN) model for hyperspectral image classification, the first time that a model had considered the intrinsic sequential data structure of a hyperspectral pixel. Therefore, the deep learning algorithm has great potential to process spectral information, both in theory and in practical applications.

The specific objectives of this study were as follows: (1) to explore the possibility of using spectral techniques to classify rice seeds that had different QTLs for BB resistance; (2) to introduce the deep learning algorithm into a protocol to identify BB resistant rice seeds with different data processing methods and to visualize the classification results; (3) to select the best discrimination models by comparing different machine learning approaches and with different spectral information as input.

## Materials and Methods

### Sample Preparation

Three major QTLs for BB resistance had previously been identified using a segregating F2 population by the State Key Laboratory Breeding Base for Zhejiang Sustainable Pest and Disease Control, Hangzhou, China (Han et al., 2014). The three QTLs were mapped on chromosomes 1, 3, and 5 and were named, respectively as qR1, qR3, and qR5. All of these QTLs had a strong effect on resistance explaining about 21.5%, 12.3%, and 39.2% of the resistance variance, respectively (Han et al., 2014). Two of those QTLs (qR3 and qR5) had been used to create the varieties of rice seed used in this study.

**FIGURE 1:**
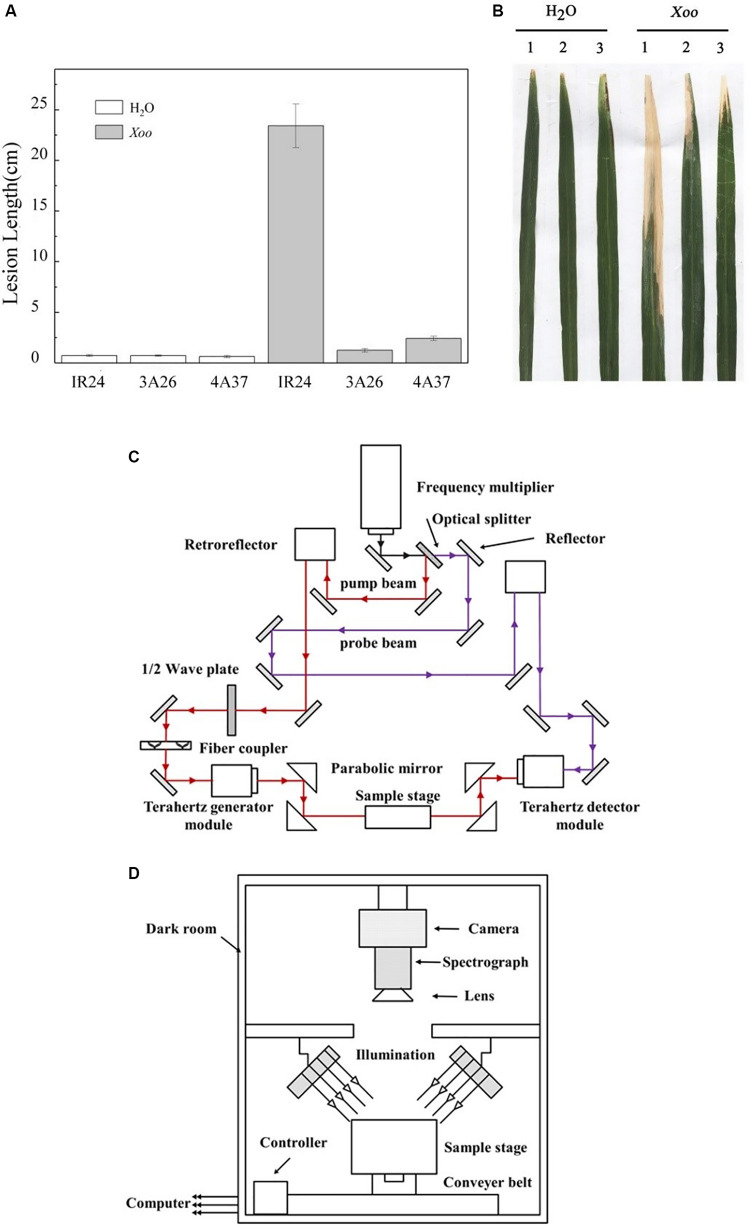
The resistance to rice bacterial blast of IR24, 3A26, and 4A37 and a schematic diagram of the optical system. Rice was inoculated by the leaf-clipping method and at least 16 leaves from four plants were inoculated. Lesion length was measured 3 weeks after inoculation. **(A)** Lesion length after mock inoculation with H2O (control) and Xoo inoculation. Bars indicate the standard error; **(B)** The resistance phenotypes of the leaves of IR24, 3A26, and 4A37. 1: IR24, 2: 3A26, and 3: 4A37; **(C)** Schematic diagram of the terahertz optical system; **(D)** Diagram showing the structure of the hyperspectral imaging system.

*Oryza sativa* ssp. indica (cv. IR24) is an elite rice cultivar developed by the International Rice Research Institute but which is highly susceptible to BB. CSSLs had been constructed by introducing the QTLs for BB resistance into IR24 under the assistance of molecular makers. Thus, line 3A26 contains qR5 and line 4A37 contains qR3 but their genetic base is the same as IR24 except for the Chromosome Single Segment containing the QTL for BB resistance. The results of a resistance test are shown in Figures 1A,B.

A total of 1980 rice seeds of the three different types (IR24, 3A26, and 4A37) were collected with the help of the State Key Laboratory Breeding Base for Zhejiang Sustainable Pest and Disease Control, Hangzhou, China. In order to eliminate moisture interference in the terahertz spectral acquisition experiment, all the samples were placed in a 40°C drying oven for 6 h before the experiment. No other additional processing was performed on those rice seeds. There were almost no differences in their shape and size.

### Terahertz Imaging System and NIR Hyperspectral Imaging System

A THz time-domain imaging system (CCT-1800; Huaxun Ark Technology Corp., Shenzhen, China) was used in this experiment. The system has four main parts: the optical part, the hardware part, the structural part and the software part. The detail of the terahertz optical system structure is shown in Figure 1C. The CCT-1800 spectrometer used a 780 nm femtosecond laser to excite semiconductor devices to generate and receive terahertz signals. The spectrometer used a voice coil motor to obtain a real-time sampled signal and a slow sweep motor to compensate for differences in optical path difference. The terahertz spectra in the range of 0.06–4 Thz could be detected and recorded by the system. The terahertz absorption spectral image of a maximum scanning area of 50 × 50 mm was obtained by using the transmission imaging module. The step size of the data acquisition was set to 0.2 mm resulting in a point-to-point scan time of 90 min per absorption spectral image. Samples were fixed on the metal frame of the carrier using Scotch tapes, and the reference signal was the terahertz absorption spectral image of the blank tape.

The structure of the NIR hyperspectral imaging system is shown in Figure 1D. The imaging spectrograph (ImSpector N17E; Spectral Imaging Ltd., Oulu, Finland) and high-performance CCD camera (C8484-05; Hamamatsu, Hamamatsu City, Japan) coupled with a camera lens (OLES22; Specim, Spectral Imaging Ltd., Oulu, Finland) were the key parts of the system. The other equipment is described in detail in Feng’s study (Feng et al., 2017). Samples were placed on the sample stage, which was a black plate on the conveyer belt. In order to obtain a clear hyperspectral image (320 × 320 × 256) with a resolution of 5 nm, the distance from the lens to the sample stage was set to 26 cm, and the exposure time of the camera was set to 3 ms. The conveyer motor controlled the speed of the conveyer belt at 23 mm/s for stable images. After acquisition by the computer, the hyperspectral images were corrected to calculate the effects of the white and black reference using the equation described by Nie et al. (2019).

### Spectral Data Collection and Pretreatment

Both the terahertz and the NIR hyperspectral imaging systems provided spectral data in the form of a data cube. This made it possible to process the data in two ways: one dimensional (1D) spectral data contained all the spectral information of each sample, while the two dimensional (2D) spectral image contained both spectral and spatial information. Of the terahertz absorption spectral data, only that in the range of 0.3–2 Thz was reliable because of the device restriction. Similarly, NIRS data outside the range of 975.01–1645.82 nm was discarded before processing to avoid the influence of the external environment and camera performance.

Regions of interests (ROI) corresponding to the shape of each rice seed sample were chosen as the extraction target before later data processing to reduce the interference of background signals. In order to obtain 1D spectral data, all the data of pixels in the ROI were averaged. The ROI used to extract the 2D spectral image was the smallest rectangular area that could contain the rice seed samples. To process the 1D terahertz absorption spectral data for baseline correction and noise cancelation, the adaptive iteratively reweighted Penalized Least Squares (airPLS) method was used with parameter λ set to 100 and the moving average (MA) window set to 3 (Zhang et al., 2010). The 1D NIRS data was also freed from noise suppression by using MA with the parameter setting of 3.

### Data Analysis Methods

Traditional machine learning algorithms including SVM, PLS-DA, and RF algorithm and deep learning algorithms like the CNN were used to classify the different varieties of rice seeds and to identify those resistant to BB.

#### Convolutional Neural Network

**FIGURE 2:**
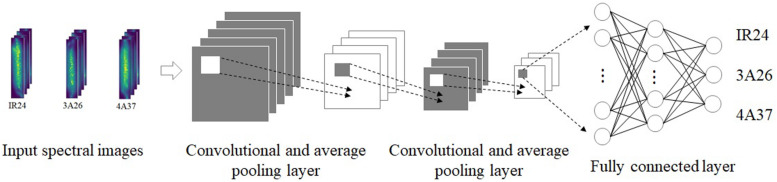
The diagram of the established CNN structure.

The CNN algorithm essentially implements the input-to-output mapping by extracting features and reducing dimensions of the data (Gu et al., 2015; Voulodimos et al., 2018). Parameter sharing and sparse links of layers help it better handle image information, which also helps in the classification of the terahertz and NIRS images. Four different structures of CNN were built to process both the 1D and 2D data from the two types of imaging system. Before feeding spectral data into the structure, normalization was performed to accelerated the convergence. The parameters chosen as giving the best performance after preliminary experiments The parameters and the structure which were chosen at the best performance after experiments could be found in Table 1 and Figure 2. There were two convolutional layers, one average pooling layer and two fully connected layers in all four CNN structures. Rectified linear units (ReLUs) were applied between those layers to avoid gradient disappearance and to make the whole discriminant model more flexible to deal with non-linear data. The equation for the ReLUs can be found in Nie’s study (Nie et al., 2019). For multiple classification, the softmax function was chosen to convert the output of the whole model into a class probability (Hinton et al., 2012). Thus the cost function, which is the cross entropy function C in equation (1), was able to measure the discrepancy between the targets d and outputs p for each sample j.

$$\text{C}=-\sum_{j}d_{j}\,\log p_{j}$$

**TABLE 1:** The parameters of CNN structures.

Structures	1D terahertz absorption spectrums	1D near-infrared hyperspectral spectrums	2D terahertz absorption image	2D near-infrared hyperspectral image
F	128	128	64	128
F2	64	64	32	64
R1	0.01	0.01	0.05	0.05
R2	0.01	0.01	0.05	0.05
KS1	2 × 1	2 × 1	2 × 2	2 × 2
KS2	2 × 1	2 × 1	2 × 2	2 × 2
AP	2 × 1	2 × 1	2 × 2	2 × 2
D1	256	256	512	64
D2	3	3	3	3
T	200	200	120	120

*F1 and F2 mean the number of filters in the first and second convolutional layer; R1 and R2 mean the L2 regularization number of the first and second convolutional later; KS1 and KS2 mean the size of filters in the first and second convolutional layer; AP means the size of filter in the average pooling layer; D1 and D2 mean the number of neurons in the first and second fully connected layer; T means the number of training epoch.*

Instead of using all the samples at once to train the CNN models, the mini-batch training method was applied to improve the speed and performance. After randomly dividing the samples into a 70% training set and a 30% validation set, only 256 random samples from the training set were sent to the model during each training. Both 1D NIR hyperspectral spectra and 2D NIR hyperspectral images were standardized for pretreatment. The adaptive moment estimation gradient descent algorithm with a learning rate of 0.001 was used to update the trainable parameters to minimize the cost function. The input sizes of 2D terahertz absorption images and 2D NIR hyperspectral images were set to 39 × 9 and 13 × 4, respectively.

#### Traditional Machine Learning Methods

As a linear classification method, PLS-DA is based on the principle of PLSR which uses projection of variables to maximize the covariance (Paul et al., 2019). In this study, only the average spectra of samples were considered as the X variables, and the Y variables were determined by a code rule such that IR24 was code 1, 3A26 was code 2 and 4A37 was code 3. After dividing the samples into training and testing sets with a ratio of 7:3, pretreatment like standardization was only performed on the NIR hyperspectral spectra. Cross-validation was used to get a relatively high and stable accuracy rate.

Support vector machine based on the margin maximization principle has been applied as a particular linear classifier (Kumar and Gopal, 2009). For the multiple classification tasks in this study, several SVMs were built to separate one class from the rest by constructing a hyperplane in a higher dimension. Radial basis function (RBF) was chosen to be the kernel function for its excellent ability to deal with non-linearity classification and the values of the hyperparameter (C, σ) were determined by the grid search method to be (1e6, 0.01) in the terahertz absorption spectrum classification and (1e5, 0.0001) for the NIR hyperspectral spectrum classification. The 10-fold cross-validation was performed after dividing the training set and testing set in a ratio of 7:3.

An ensemble of decision tree classifiers which are generated using the random vectors sampled independently from the input vectors constitute the RF classifier (Pal, 2005). Each tree classifier is supposed to count as an independent unit for the most popular class, and the classification results are aggregated and averaged to provide the output of the RF (Svetnik et al., 2003). In this study, the number of sub-data sets which were generated by sampling using the replacement method with the original data set was set to 10. By using 10-fold cross-validation, the maximum number of features when building the optical decision tree was set to 14. NIRS spectra were standardized before feeding into the model.

### Data Visualization

In order to visualize the progress of classification of the 1D spectral data processed by the deep learning algorithm and intuitively understand the effectiveness of the deep learning algorithm in extracting features, we used t-SNE to perform non-linear dimensionality reduction on the feature data. Every datapoint of the high-dimensional feature map was given a location in a two-dimensional map. It has been shown that t-SNE is a powerful tool to create a single map that can reveal structure at many different scales (Laurens and Hinton, 2008). The perplexity of the t-SNE algorithm was set to 30 and the maximum number of iterations was set to 500 before reducing the dimensions of features into two dimensions.

### Software Tools

All data were processed on a computer with a Win10 64-bit operating system, with Inter(R) Core(TM) i5-7500 CPU, 3.40 GHz and 8 GB RAM. A deep learning framework Keras^1^ was used to construct the CNN and we found that both the spectral data and spectral images could be processed quickly. Average spectra were extracted with the help of MATLAB R2013b. Traditional machine learning algorithms and t-SNE were implemented using the program language Python 3.6^2^ on the Jupyter Notebook web-based application for interactive computing^3^. Graphical work was done using Pro 9.0 (Origin Lab Corporation, Northampton, MA, United States).

## Results

### Spectroscopic Results

**FIGURE 3:**
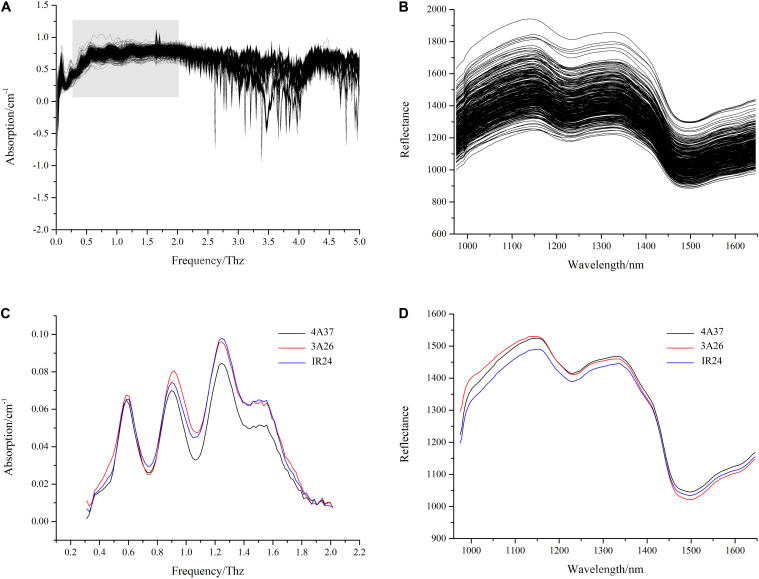
The average spectra of samples. **(A)** the original terahertz absorption spectra; **(B)** the extracted and preprocessed terahertz absorption spectra; **(C)** the original near-infrared hyperspectral spectra; **(D)** the extracted and preprocessed near-infrared hyperspectral spectra.

The average terahertz absorption spectra of the seeds of the three different lines were extracted from the original absorption spectra in the frequency range of 0.3–2 THz (Figures 3A,B). After preprocessing, it was clear that, although the absorption curves were generally similar in shape as might be expected, there were some differences between the samples at frequencies around 0.9 THz, 1.1 THz, and 1.3 THz. Similar results were observed in transgenic sugar beet (Liu et al., 2015). The spectral information in the range of 975.01–1645.82 nm (Figures 3C,D) show that the seeds of the BB resistant lines 4A37 and 3A26 had very similar curves but showed some clear differences to the that of the original cultivar IR24.

### Classification Results

**FIGURE 4:**
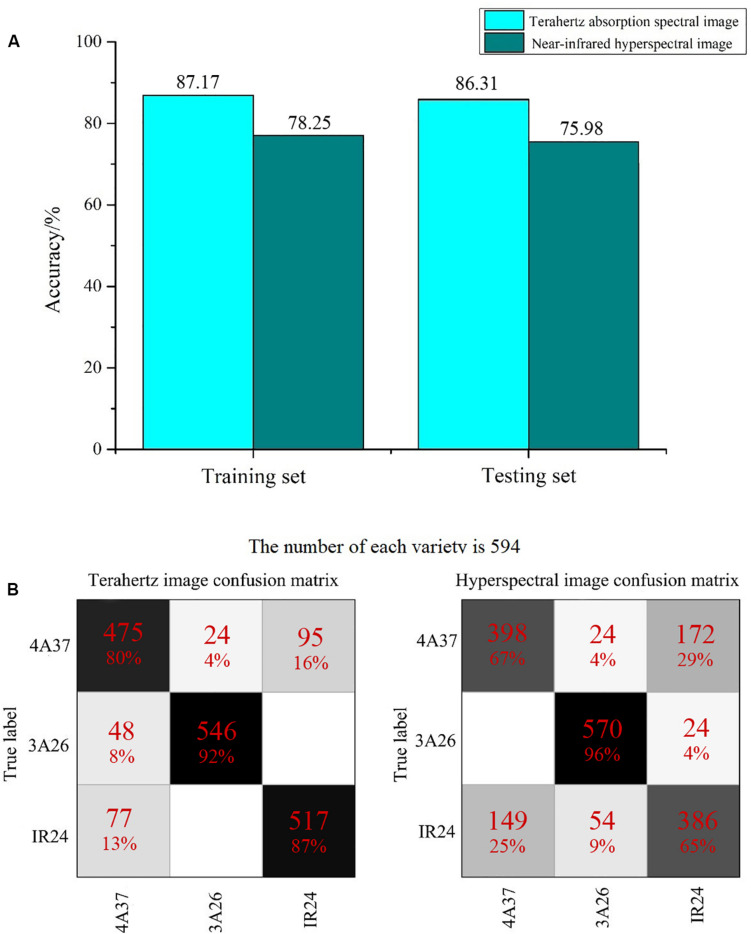
The classification results using 2D spectral images of rice seeds. **(A)** Histograms showing the overall accuracy of classification; **(B)** Confusion matrixes exploring the classification results in detail.

The first objective was to explore the effectiveness of using 2D spectral images for reliable identification. The reliability of classification in this study using terahertz absorption spectral images and NIR hyperspectral images together with CNN is shown in Figure 4A. The classification of rice seeds using terahertz absorption spectral images had an accuracy of 86.31%, about 10% higher than when using NIR hyperspectral images. In studies using larger samples Qiu et al. (2018) found that the classification of seeds of four rice varieties was only 87% accurate using the NIR spectrum extracted from hyperspectral imaging in the special spectral range combined with CNN. Examination of the confusion matrixes (Figure 4B) suggests that the poor classification obtained using NIR occurred because the CNN model did not clearly distinguish 4A37 from IR24, whereas this problem was much less in analyses of the terahertz spectral images.

**FIGURE 5:**
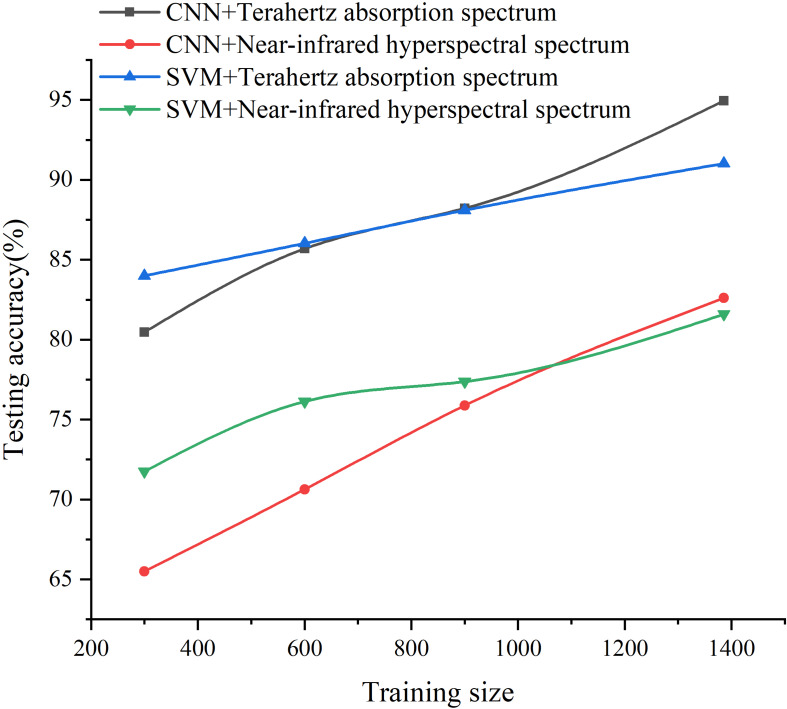
The impact of the training size on the testing accuracy of 1D spectra based on CNN and SVM.

The 1D spectra of the rice seeds, which concentrate the spectral information of each pixel of each sample and remove the interference of background noise, were next used as inputs for different discriminant models including CNN, SVM, PLS-DA, and RF. As shown in Table 2, discriminant models based on CNN and SVM had similar accuracy on both training and testing sets. CNN provided about 10% more accurate classification using the 1D spectra (Table 2) than the 2D spectral images (Figure 4A). The impact of training set size on the classification accuracy based CNN and SVM using 1D spectra had been studied (Figure 5). It was evident that with the increase of the training size, the identification performance of CNN and SVM established by using both terahertz spectrum and near-infrared hyperspectral spectrum was improved a lot. The same results as in Table 2 could be obtained that models based on terahertz spectrum outperform the models based on NIR hyperspectral spectrum. And there were distinct crossing points on the curves of CNN and SVM models, before which the accuracies of SVM models were higher than the accuracies of the CNN models.

**TABLE 2:** Classification accuracy using 1D spectra and different discriminant models.

Discriminant model	Data resource	The accuracy of training set	The accuracy of testing set
CNN	Terahertz absorption spectrum	97.50%	94.95%
SVM	Terahertz absorption spectrum	95.60%	91.02%
RF	Terahertz absorption spectrum	83.28%	81.03%
PLS-DA	Terahertz absorption spectrum	58.62%	54.99%
CNN	Near-infrared hyperspectral spectrum	88.57%	82.60%
SVM	Near-infrared hyperspectral spectrum	86.30%	81.59%
RF	Near-infrared hyperspectral spectrum	62.58%	60.72%
PLS-DA	Near-infrared hyperspectral spectrum	48.28%	42.70%

**FIGURE 6:**
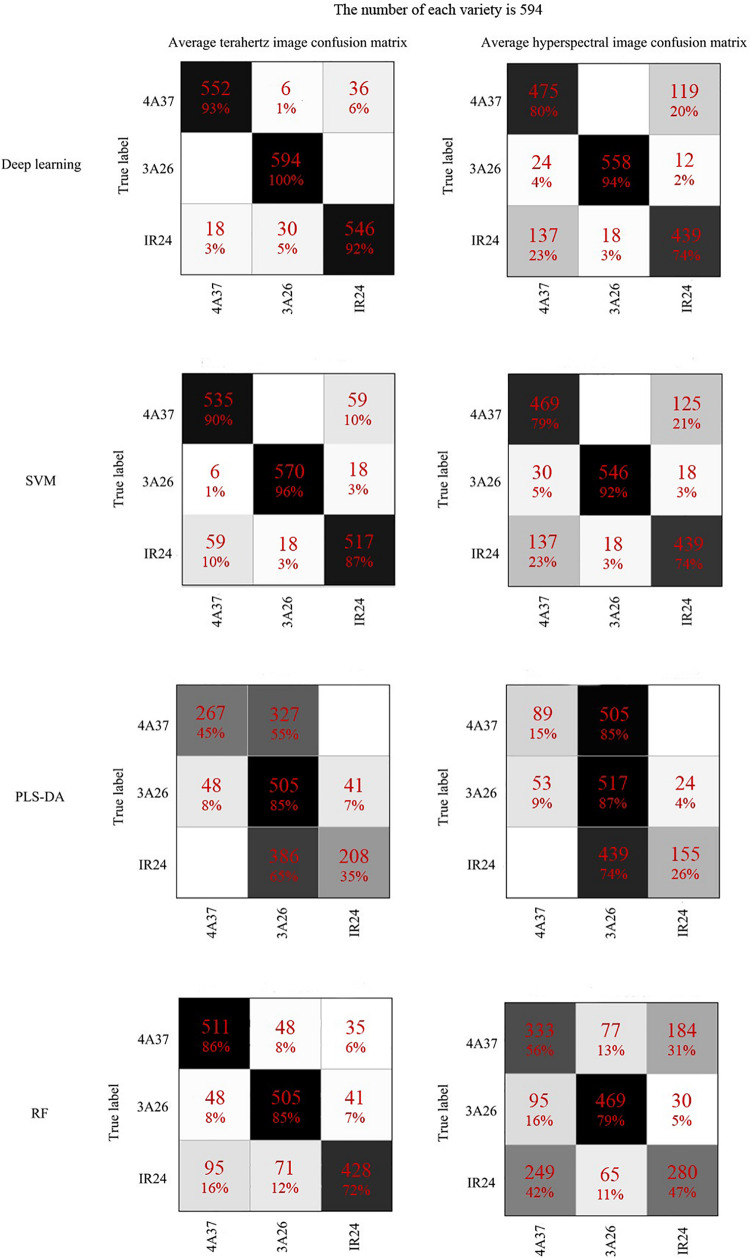
The confusion matrixes of the classification results of 1D spectra based on different discriminant models.

Table 2 also showed that there was no severe model overfitting. The use of RF to classify terahertz spectra was less accurate (about 81.03%) than CNN or SVM but better than the results from PLS-DA. The detailed analysis presented in Figure 6 shows, that as in Figure 4, seeds of 3A26 had the highest correct recognition rate in almost all the confusion matrices, while the probabilities of misclassifying 4A37 and IR24 were relatively high. And also based on the results of those classification indicators in Table 3, apparently the specificity, sensitivity and F-score of each variety were relatively higher while spectral images were applied. When it came to seeds of 3A26, better performance than the other two varieties were achieved in these classification measures using CNN, SVM and RF with all lower results acquired from the PLS-DA models.

**TABLE 3:** The comparison of classification results from different studies.

Study object	Discriminant model	Data resource	Classification accuracy	Source
Bacterial blight resistant rice seed	CNN	Terahertz absorption spectrum	94.95%	
Transgenic rice seed	RF	Terahertz reflection spectrum	95%	Hu et al., 2017
Transgenic rice seed	SVM	Terahertz absorption spectrum	92.08%	Lian et al., 2017
Rice false smut seed	RF-ELM	Near-infrared hyperspectral spectrum	91.07%	Wu et al., 2020
DON contaminated wheat	LDA	Near-infrared hyperspectral spectrum	62.7%	Femenias et al., 2020
Maize seeds	RBFNN	Near-infrared hyperspectral spectrum	91.00%	Zhao et al., 2018

### Visualization Results

The visualization of processed data which has a wide range of dimensional changes has become an important issue in many fields (Laurens and Hinton, 2008). Visualization allows us to clearly understand the process of data processing and partly resolves the black box mystery of modeling algorithms like deep learning.

**FIGURE 7:**
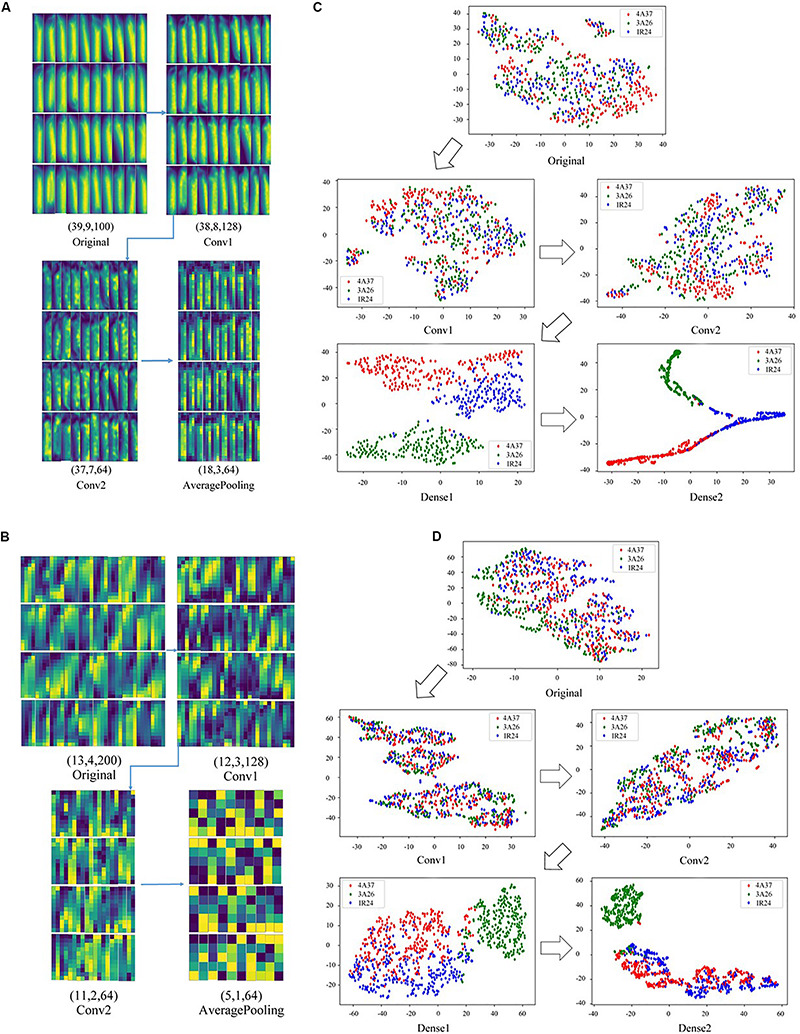
The feature maps of different models and visualization maps based on t-SNE of the model output. **(A)** The feature maps of the first and second convolutional layers of 2D terahertz absorption images based on CNN; **(B)** The visualization maps of CNN output of each layer using terahertz absorption spectra; **(C)** The feature maps of the first and second convolutional layers of 2D near-infrared hyperspectral images based on CNN; **(D)** The visualization maps of CNN output of each layer using near-infrared hyperspectral spectra.

Figure 7A presents the changes in the feature maps which are the first and second convolutional layers of the CNN in the classification of 2D terahertz absorption images. As the size of the feature map and the number of image channels (which represent different frequencies) decreased, the discriminant model was still able to focus on the main part of the seed. Also, before it was stretched and sent to the fully connected layer, the last visualization map still had the approximate shape of rice seeds, showing that the main spectral information had been retained. By contrast, the NIR hyperspectral images of the rice seeds had lost their original shape after standardization (Figure 7C). However, pre-experiments had shown that standardization of NIR hyperspectral images improved the accuracy of classification so it possible that the shape of rice seeds was not the key factor for their classification. In Figure 7C, the hyperspectral image was finally sent to the full connected layer with only 5 spectral pixels which was much fewer than those in Figure 7A. Apart from the differences of spectral technology, the differences in the number of pixels that contain spectral information might be another decisive factor determining the accuracy of classification of these 2D images.

A non-linear dimensionality reduction method, t-SNE, that simultaneously preserves the local and global structure of data was used to visualize the processes of data processing of 1D spectra based on CNN using both terahertz and NIR hyperspectral imaging technology. In Figure 7B, the data points of the rice seeds represented by different colors were processed by the CNN model using terahertz absorption spectra. As a result, those data points gradually changed from overlapping to clearly separable. Similarly, in the visualization of the CNN output of NIR hyperspectral spectra, all three class of rice seeds were distinguished by distinct boundaries in the last feature map in Figure 7D. Moreover, the overlap caused by the misclassification of the data points on the last feature map was more serious than that of Figure 7B.

## Discussion

In the present study, spectral technologies established with different characteristics were applied to determine the class of BB resistance rice seeds. As a non-destructive method for qualitative and quantitate analysis, both terahertz spectral technology and hyperspectral technology play an important role. When the basic evidence for the effectiveness of spectral technology is considered, the relationship between the sample composition and spectra is the most fundamental. It is known that the stretching vibration of several hydrogen-containing functional groups that chemical bond organic molecules (e.g., C-H, O-H, N-H) are related to the absorption band of NIR hyperspectral spectra (Serranti et al., 2013). Ultimately, phenotypic changes are the best indicator of genotypic structural changes (Munck et al., 2004) but those changes will inevitably involve molecular changes. Therefore, the different distances between the average curves in Figure 3 may suggest that the combined effects of the stretching vibration of the hydrogen-containing functional groups in the different spectral bands was changeable which probably affected the accuracy of classification using different modeling algorithms. It is worth mentioning that most biomolecules such as DNA and protein can be detected in the terahertz absorption spectrum.

Plenty of studies provide a solid foundation for the effectiveness of applying spectroscopy, but only a few of them investigate the performance of spectral images instead of spectrums in the field of plant and food detection. These 2D images make full use of the spectral and spatial information with more channels than a normal photographic image which has only three channels. It has already been shown that deep learning methods like CNN have advantages in utilizing the features of spectral images (Wang et al., 2017; Wu and Prasad, 2017). As shown in Figure 4, those classification results indicated the performance of NIR hyperspectral imaging technology for obtaining internal information from rice seeds varies on different testing samples. The use of 2D spectral images of rice seeds combined with deep learning algorithms shows great potential for classifying rice seeds which are genetically very similar but with Chromosome Single Segment Substitutions. The results (Table 2) showed that for each algorithm, much more accurate classification was obtained using the terahertz absorption spectra than the NIR ones, perhaps because the terahertz spectra can detect biomolecules (Kistner et al., 2007; Hu et al., 2017). The variation in the output of each layer on the visualization map (Figure 7) showed the effectiveness of the deep learning algorithm for identifying the terahertz absorption spectra of the BB resistant rice seeds. Compared with feature maps of NIR spectral data, the feature maps of terahertz spectra were much more districted and the terahertz absorption spectra provided the better input for establishing a discriminant model based on the deep learning algorithm. From the comparison of the results from relevant studies in Table 3, it could be found that different spectral techniques coupled with different models can lead to various identification performance. Lian et al. (2017) also used SVM to identify transgenic ingredients in maize using terahertz spectra and reported relatively high (>90%) accuracy. Chen et al. (2017) reported similar accuracy of classification when comparing deep learning and SVM models used with spatially dominated hyperspectral data. The SVM model builds its decision boundary based on the data points that are difficult to classify while CNN also has a powerful ability to utilize the deep features of the spectra (Wakholi et al., 2018). In addition, SVM algorithm has a strong ability to process small amounts of data while the deep learning algorithm requires a large amount of data (Kumar and Gopal, 2009) but in our study they were trained using the same amount of data which might be the cause of the similar classification results on the 1D spectra. From the results in Figure 5 it could be observed that with the increase of training set size, the performance of CNN models continued to improve while the SVM models kept a relative stable state. This curvilinear trend is exact the essence of the advantage of deep learning algorism that the more data the model can get and the higher accuracy the model can perform. However, when it comes to hyperspectral data classification, smaller training size is also available in the tensor-based learning (Makantasis et al., 2018). Makantasis et al. (2018) proposed a tensor-based non-linear model that was applied to classify hyperspectral images to achieve high accuracy with some amount of samples. And it can be an appropriate method to avoid the problem of lacking enough spectral data of training samples.

A multi-level features extraction method to track the target in remote sensing images was published by Zhou et al. (2020) for improving the monitoring performance. Compared to the studies of multi-level features extraction method, one of the distinctive advantage of deep learning algorithm like CNN is that the structure is able to extract and keep the critical features which can benefit the model most automatically without complex feature extraction procedures. However, taking into account the limitations of experimental equipment the CNN classification results using 1D spectral data (Table 2) were better than those using 2D images (Figure 4), because the cropping of the original spectral images resulted in the loss of the spatial information and the low resolution of the input spectral images (Figures 7A,B) and the background noise affected the performance of the discriminant model. With enough information of spectral pixels, even 100% accuracy could be obtained in the study of fish filet identification using different classifiers (Qin et al., 2020). Furthermore, irregular topographies of the seeds spectral images which leads to the reflectance changes at different image position is also a vital factor causing bad performance of the models. Considering the situation, averaging spectral images became a method of avoiding the changes and integrating information. And certainly there are other methods to make better use of the abundant information contained in spectral images. Yuan et al. (2020) had studied a new method which provided another path to improve the classification of spectral images apart from averaging the images to correct the spectral reflectance.

As for the other traditional machine learning algorithms, RF is an efficient classifier that can operate on large datasets and in this study there may not have been enough spectra for RF to reach its full potential (Rodriguez-Galiano et al., 2012). The method of selecting hyperparameters also affected the performance of RF in the classification of these rice seeds. PLS-DA is a linear classifier and has already proved inadequate to deal with spectral data that has many features to be considered in the classification (Nie et al., 2019). Similarly, only 62.7% recognition rate was obtained in the classification of deoxynivalenol (DON) contaminated wheat kernels using linear classifier based on hyperspectral imaging technology, which showed the unqualified identification ability of the linear classifier (Femenias et al., 2020). Moreover the PLS-DA model always tends to establish decision boundaries based on easily classified data points, resulting in misclassification of outliers (Wakholi et al., 2018). The samples used in this study were genetically very closely related, being based on the same rice variety but with different QTLs selected. As shown in Table 4, classification measures varied widely for different varieties. However, the seeds of 3A26 had kept a relatively high value in the specificity, sensitivity and F-score which indicated that those discriminant models were able to accurately identify the 3A26. The performance of different discriminant models established by using distinct algorithms were also reflected in the classification measures verifying the classification results in a more detailed perspective. It seems likely that the biomolecules which are expressed by the genes of 4A37 and IR24 are more similar to one another than they are to those of 3A26. Qin et al. (2020) also found that there was misclassification in the classification of fish freshness and they believed that might be related to the condition of the fish samples which had a progressive change in the tissue. This gives some direction for further studies of the essential differences between these rice seeds related to BB resistance.

**TABLE 4:** The related classification indicators of each discriminant model.

Data resource	Discriminant model	Variety	Specificity	Sensitivity	F-score
Terahertz absorption image	CNN	4A37	0.895	0.800	0.796
		3A26	0.980	0.920	0.939
		IR24	0.920	0.870	0.857
Near-infrared hyperspectral image	CNN	4A37	0.874	0.670	0.698
		3A26	0.935	0.960	0.919
		IR24	0.835	0.650	0.657
Terahertz absorption spectrum	CNN	4A37	0.985	0.930	0.949
		3A26	0.970	1.000	0.971
		IR24	0.970	0.920	0.930
Near-infrared hyperspectral spectrum	CNN	4A37	0.865	0.800	0.773
		3A26	0.985	0.940	0.954
		IR24	0.890	0.740	0.755
Terahertz absorption spectrum	SVM	4A37	0.945	0.900	0.896
		3A26	0.985	0.960	0.965
		IR24	0.935	0.870	1.000
Near-infrared hyperspectral spectrum	SVM	4A37	0.860	0.790	0.763
		3A26	0.985	0.920	0.944
		IR24	0.880	0.740	0.747
Terahertz absorption spectrum	PLS-DA	4A37	0.960	0.450	0.588
		3A26	0.400	0.850	0.557
		IR24	0.965	0.350	0.528
Near-infrared hyperspectral spectrum	PLS-DA	4A37	0.955	0.150	0.242
		3A26	0.205	0.870	0.503
		IR24	0.980	0.260	0.400
Terahertz absorption spectrum	RF	4A37	0.880	0.860	0.819
		3A26	0.900	0.850	0.829
		IR24	0.935	0.720	0.778
Near-infrared hyperspectral spectrum	RF	4A37	0.710	0.560	0.523
		3A26	0.880	0.790	0.778
		IR24	0.820	0.470	0.514

All in all, this study used both terahertz and NIR hyperspectral imaging to obtain spectral information from rice seeds that were genetically very similar apart from specific QTLs for BB resistance. A deep learning method (CNN) and traditional machine learning methods (SVM, PLS-DA, and RF) were applied to build discriminant models based on either the 2D spectral images or the 1D spectra. In all tests, using the terahertz absorption spectra provided better discriminant models than were obtained with NIR hyperspectral spectra. The CNN and SVM models outperformed the other models in accuracy of classification accuracy, reaching around 91% with 1D spectra. The steps in extracting CNN features of 2D spectral images were visualized and the results indicate that the size of the image, which also represents the quantity of the spectral pixels, may affect the classification. The t-SNE visualization provided a particularly vivid way to observe the process of CNN processing of the spectra. Therefore, terahertz imaging technology combined with CNN in this study can provide a powerful method for plant breeders to quickly identify BB resistant samples. In the future, rice samples with different labeled chromosomes could be studied to build a classification database based on CNN or other deep learning algorithms.

## Data Availability Statement

The raw data supporting the conclusions of this article will be made available by the authors, without undue reservation, to any qualified researcher.

## Author Contributions

JZ, YY, XF, and HX performed the measurements. JZ and YY wrote the manuscript. JZ, YY, JC, and YH designed the experiment. JC and YH reviewed the initial design of the experiments and made guidance for the writing of the manuscript. All authors reviewed the manuscript.

## Conflict of Interest

The authors declare that the research was conducted in the absence of any commercial or financial relationships that could be construed as a potential conflict of interest.

## Footnotes

1 https://keras.io/zh/

2 https://www.python.org/

3 https://jupyter.org/

## Abbreviations

BB: bacterial blight
CNN: convolutional neural network
ELM: extreme learning machine
LDA: linear discriminant analysis
PLS-DA: partial least squares discriminant analysis
RBFNN: radial basis function neural network
RF: random forest
SVM: support vector machine
t-SNE: t-distributed stochastic neighbor embedding.

## References

[B1] Chen Y. Lin Z. Xing Z. Gang W. Gu Y. (2017). Deep learning-based classification of hyperspectral data. *IEEE J. Sel. Top. Appl. Earth Observ. Remote Sens.* 7 2094–2107. 10.1109/JSTARS.2014.2329330

[B2] Cui Y. Ge W. Li J. Zhang J. An D. Wei Y. (2019). Screening of maize haploid kernels based on near infrared spectroscopy quantitative analysis. *Comput. Electron. Agric.* 158 358–368. 10.1016/j.compag.2019.01.038

[B3] Demidova E. V. Goryachkovskaya T. N. Mescheryakova I. A. Malup T. K. Peltek S. E. (2016). Impact of terahertz radiation on stress-sensitive genes of *E.coli* cell. *IEEE Trans. Terahertz Sci. Technol.* 6 435–441. 10.1109/TTHZ.2016.2532344

[B4] Femenias A. Gatius F. Ramos A. J. Sanchis V. Marín S. (2020). Standardisation of near infrared hyperspectral imaging for quantification and classification of DON contaminated wheat samples. *Food Control.* 111:107074 10.1016/j.foodcont.2019.107074

[B5] Feng X. Peng C. Chen Y. Liu X. Feng X. He Y. (2017). Discrimination of CRISPR/Cas9-induced mutants of rice seeds using near-infrared hyperspectral imaging. *Sci. Rep.* 7:15934. 10.1038/s41598-017-16254-z 29162881 PMC5698449

[B6] Gu J. Wang Z. Kuen J. Ma L. Shahroudy A. Bing S. (2015). Recent advances in convolutional neural networks. *Pattern Recognit.* 77 354–377. 10.1016/j.patcog.2017.10.013

[B7] Guo Y. Liu Y. Oerlemans A. Lao S. Wu S. Lew M. S. (2016). Deep learning for visual understanding: a review. *Neurocomputing* 187 27–48. 10.1016/j.neucom.2015.09.116

[B8] Gutierrez S. Fernandez-Novales J. Diago M. P. Tardaguila J. (2018). On-The-Go hyperspectral imaging under field conditions and machine learning for the classification of grapevine varieties. *Front. Plant Sci.* 9:1102. 10.3389/fpls.2018.01102 30090110 PMC6068396

[B9] Han X. Yang Y. Wang X. Zhou J. Zhang W. Yu C. (2014). Quantitative trait Loci mapping for bacterial blight resistance in rice using bulked segregant analysis. *Int. J. Mol. Sci.* 15 11847–11861. 10.3390/ijms150711847 24995697 PMC4139818

[B10] Hinton G. Deng L. Yu D. Dahl G. E. Mohamed A. R. Jaitly N. (2012). Deep neural networks for acoustic modeling in speech recognition: the shared views of four research groups. *IEEE Signal. Process. Mag.* 29 82–97. 10.1109/MSP.2012.2205597

[B11] Hu X. Lang W. Wei L. Xue X. Yang J. Lei Z. (2017). A non-destructive terahertz spectroscopy-based method for transgenic rice seed discrimination via sparse representation. *J. Infrared Millim. Terahertz Waves.* 38 1–12. 10.1007/s10762-017-0392-z

[B12] Jaganathan D. Ramasamy K. Sellamuthu G. Jayabalan S. Venkataraman G. (2018). CRISPR for crop improvement: an update review. *Front. Plant Sci.* 9:985. 10.3389/fpls.2018.00985 30065734 PMC6056666

[B13] Kistner C. André A. Fischer T. Thoma A. Janke C. Bartels A. (2007). Hydration dynamics of oriented DNA films investigated by time-domain terahertz spectroscopy. *Appl. Phys. Lett.* 90:233902 10.1063/1.2743401

[B14] Kumar M. A. Gopal M. (2009). Least squares twin support vector machines for pattern classification. *Expert Syst. Appl.* 36 7535–7543. 10.1016/j.eswa.2008.09.066

[B15] Laurens V. D. M. Hinton G. (2008). Visualizing data using t-SNE. *J. Mach. Learn. Res.* 9 2579–2605.

[B16] Lian F. Xu D. Fu M. Ge H. Yuan Z. (2017). Identification of transgenic ingredients in maize using terahertz spectra. *IEEE Trans. Terahertz Sci. Technol.* 7 378–384. 10.1109/TTHZ.2017.2708983

[B17] Liu J. (2017). Determination of transgenic organisms from non-transgenic using terahertz spectroscopy and chemometrics. *Optik* 131 885–891. 10.1016/j.ijleo.2016.11.213

[B18] Liu J. Li Z. Hu F. Chen T. Du Y. Xin H. (2015). Identification of transgenic organisms based on terahertz spectroscopy and hyper sausage neuron. *J. Appl. Spectrosc.* 82 104–110. 10.1007/s10812-015-0071-6

[B19] Liu W. Liu C. Hu X. Yang J. Zheng L. (2016). Application of terahertz spectroscopy imaging for discrimination of transgenic rice seeds with chemometrics. *Food Chem.* 210 415–421. 10.1016/j.foodchem.2016.04.117 27211665

[B20] Liu P. Zhang H. Eom K. B. (2016). Active deep learning for classification of hyperspectral images. *IEEE J. Sel. Top. Appl. Earth Observ. Remote Sens.* 10 712–724. 10.1109/JSTARS.2016.2598859

[B21] Makantasis K. Doulamis A. D. Doulamis N. D. Nikitakis A. (2018). Tensor-based classification models for hyperspectral data analysis. *IEEE Trans. Geosci. Remote Sens.* 56 6884–6898. 10.1109/TGRS.2018.2845450

[B22] Mou L. Ghamisi P. Xiao X. (2017). Deep recurrent neural networks for hyperspectral image classification. *IEEE Trans. Geosci. Remote Sens.* 55 3639–3655. 10.1109/TGRS.2016.2636241

[B23] Mueller-Holtz M. Seker H. Smith G. (2014). Wavelet denoising and reconstruction of a microneedle embedded in human skin ex-vivo using terahertz pulsed reflectance. *Eng. Med. Biol. Soc.* 2014 6740–6743. 10.1109/EMBC.2014.6945175 25571543

[B24] Munck L. Møller B. Jacobsen S. Søndergaard I. (2004). Near infrared spectra indicate specific mutant endosperm genes and reveal a new mechanism for substituting starch with (1→3,1→4)-β-glucan in barley. *J. Cereal Sci.* 40 213–222. 10.1016/j.jcs.2004.07.006

[B25] Nagatsuma T. Ducournau G. Renaud C. C. (2016). Advances in terahertz communications accelerated by photonics. *Nat. Photon.* 10 371–379. 10.1038/NPHOTON.2016.65

[B26] Nakajima S. Shiraga K. Suzuki T. Kondo N. Ogawa Y. (2019). Quantification of starch content in germinating mung bean seedlings by terahertz spectroscopy. *Food Chem.* 294 203–208. 10.1016/j.foodchem.2019.05.065 31126454

[B27] Nie P. C. Zhang J. N. Feng X. P. Yu C. L. He Y. (2019). Classification of hybrid seeds using near-infrared hyperspectral imaging technology combined with deep learning. *Sens. Actuator B Chem.* 296:126630 10.1016/j.snb.2019.126630

[B28] Orrillo I. Cruz-Tirado J. P. Cardenas A. Oruna M. Carnero A. Barbin D. F. (2019). Hyperspectral imaging as a powerful tool for identification of papaya seeds in black pepper. *Food Control* 101 45–52. 10.1016/j.foodcont.2019.02.036

[B29] Pal M. (2005). Random forest classifier for remote sensing classification. *Int. J. Remote Sens.* 26 217–222. 10.1080/01431160412331269698

[B30] Paul A. Wander L. Becker R. Goedecke C. Braun U. (2019). High-throughput NIR spectroscopic (NIRS) detection of microplastics in soil. *Environ. Sci. Pollut. Res.* 26 7364–7374. 10.1007/s11356-018-2180-2 29754299

[B31] Qin J. Vasefi F. Hellberg R. S. Akhbardeh A. Isaacs R. B. Yilmaz A. G. (2020). Detection of fish fillet substitution and mislabeling using multimode hyperspectral imaging techniques. *Food Control.* 114:107234 10.1016/j.foodcont.2020.107234

[B32] Qin J. Ying Y. Xie L. (2013). The detection of agricultural products and food using terahertz spectroscopy: a review. *Appl. Spectrosc. Rev.* 48 439–457. 10.1080/05704928.2012.745418

[B33] Qiu Z. Chen J. Zhao Y. Zhu S. He Y. Zhang C. (2018). Variety identification of single rice seed using hyperspectral imaging combined with convolutional neural metwork. *Appl. Sci. Basel.* 8:212 10.3390/app8020212

[B34] Rodriguez-Galiano V. F. Ghimire B. Rogan J. Chica-Olmo M. Rigol-Sanchez J. P. (2012). An assessment of the effectiveness of a random forest classifier for land-cover classification. *ISPRS J. Photogramm. Remote Sens.* 67 93–104. 10.1016/j.isprsjprs.2011.11.002

[B35] Serranti S. Cesare D. Marini F. Bonifazi G. (2013). Classification of oat and groat kernels using NIR hyperspectral imaging. *Talanta* 103 276–284. 10.1016/j.talanta.2012.10.044 23200388

[B36] Shasmita M. D. Mohapatra P. K. Naik S. K. Mukherjee A. K. (2019). Priming with salicylic acid induces defense against bacterial blight disease by modulating rice plant photosystem II and antioxidant enzymes activity. *Physiol. Mol. Plant Pathol.* 108:101427 10.1016/j.pmpp.2019.101427

[B37] Shen Y. C. Lo T. Taday P. F. Cole B. E. Tribe W. R. Kemp M. C. (2005). Detection and identification of explosives using terahertz pulsed spectroscopic imaging. *Appl. Phys. Lett.* 86:241116 10.1063/1.1946192

[B38] Svetnik V. Liaw A. Tong C. Culberson J. C. Sheridan R. P. Feuston B. P. (2003). Random forest: a classification and regression tool for compound classification and QSAR modeling. *J. Chem. Inf. Comput. Sci.* 43 1947–1958. 10.1021/ci034160g 14632445

[B39] Voulodimos A. Doulamis N. Doulamis A. Protopapadakis R. (2018). Deep learning for computer vision: a brief review. *Comput. Intell. Neurosci.* 2018:7068349. 10.1155/2018/7068349 29487619 PMC5816885

[B40] Wakholi C. Kandpal L. M. Lee H. Bae H. Park E. Kim M. S. (2018). Rapid assessment of corn seed viability using short wave infrared line-scan hyperspectral imaging and chemometrics. *Sens. Actuator B Chem.* 255 498–507. 10.1016/j.snb.2017.08.036

[B41] Wang L. Zhang J. Peng L. Choo K. K. R. Fang H. (2017). Spectral–spatial multi-feature-based deep learning for hyperspectral remote sensing image classification. *Soft Comput.* 21 213–221. 10.1007/s00500-016-2246-3

[B42] Wu H. Prasad S. (2017). Semi-supervised deep learning using pseudo labels for hyperspectral image classification. *IEEE Trans. Image Process.* 27 1259–1270. 10.1109/TIP.2017.2772836 29990156

[B43] Wu N. Jiang H. Bao Y. Zhang C. Zhang J. Song W. (2020). Practicability investigation of using near infrared hyperspectral imaging to detect rice kernels infected with rice false smut in different conditions. *Sens. Actuator B Chem.* 308:127696 10.1016/j.snb.2020.127696

[B44] Xu W. Xie L. Ye Z. Gao W. Yao Y. Chen M. (2015). Discrimination of transgenic rice containing the cry1ab protein using terahertz spectroscopy and chemometrics. *Sci. Rep.* 5:11115. 10.1038/srep11115 26154950 PMC4495602

[B45] Yuan D. Jiang J. Qiao X. Qi X. Wang W. (2020). An application to analyzing and correcting for the effects of irregular topographies on NIR hyperspectral images to improve identification of moldy peanuts. *J. Food Eng.* 280:109915 10.1016/j.jfoodeng.2020.109915

[B46] Yuan L. (2014). Development of hybrid rice to ensure food security. *Rice Sci.* 21 1–2. 10.1016/S1672-6308(13)60167-5

[B47] Zhang Z. Chen S. Liang Y. (2010). Baseline correction using adaptive iteratively reweighted penalized least squares. *Analyst* 135 1138–1146. 10.1039/b922045c 20419267

[B48] Zhao Y. Zhu S. Zhang C. Feng X. Feng L. He Y. (2018). Application of hyperspectral imaging and chemometrics for variety classification of maize seeds. *RSC Adv.* 8 1337–1345. 10.1039/c7ra05954j PMC9077125 35540920

[B49] Zhou B. Duan X. Ye D. Wei W. Wozniak M. Polap D. (2020). Multi-level features extraction for discontinuous target tracking in remote sensing image monitoring. *Sensors* 19:4855. 10.3390/s19224855 31703427 PMC6891746

